# Catheter Ablation of Atrial Fibrillation: A Review of the Current Status and Future Directions

**DOI:** 10.19102/icrm.2017.081101

**Published:** 2017-11-15

**Authors:** Daniel P. Melby

**Affiliations:** ^1^Minneapolis Heart Institute at Abbott Northwestern Hospital, Minneapolis, MN, USA

**Keywords:** Atrial fibrillation, catheter ablation, review

## Abstract

Atrial fibrillation (AF) is one of the most common arrhythmias encountered in clinical practice today. Over the last 20 years, the frequency of use of catheter ablation to treat AF has grown, commensurate with the rise in arrhythmia burden and via a number of technical advancements. These developments can be divided into new techniques for myocardial ablation, improvements in the understanding of AF trigger mechanisms, and advancements in atrial mapping. Progress in these fields has led to a fundamental change in daily practice, and has contributed to a rise, for ablation, from a procedure performed infrequently at select centers to one that is commonplace worldwide. In this article, the data and methods leading to this fundamental change will be presented and discussed.

## Introduction

Atrial fibrillation (AF) is one of the most common arrhythmias encountered in clinical practice. Population studies have revealed an increase in AF prevalence over the past 30 years, with projections for the observed growth to continue.^1–6^ Over the last 20 years, the frequency of catheter ablation for AF has also increased, commensurate with the rise in arrhythmia burden and via a number of technical advancements.^7,8^ These developments can be divided into three fields: new techniques for endocardial ablation, improvements in the understanding of AF trigger mechanisms, and advances in electroanatomical mapping. Progress in these areas has led to a fundamental change in daily practice, and has contributed to the evolution in ablation from a procedure performed infrequently at select centers to one that is commonly relied upon worldwide.

The current 2014 American College of Cardiology/American Heart Association guidelines for the treatment of AF position ablation as a class I option for the treatment of symptomatic drug-refractory persistent AF. Primary ablation of paroxysmal AF prior to drug failure is a class IIa recommendation.^9^ With further technical advancements, it is reasonable to anticipate expanded procedural indications, as well as improved procedural outcomes and techniques. This review will discuss the history, current status, and anticipated future developments of myocardial ablation and AF mapping techniques.

## Ablation techniques for AF

Catheter ablation for AF was first described in 1994 in combination with several ongoing endeavors, including attempts to replicate a surgical MAZE lesion set,^10^ the performance of isolated right atrial linear lesions,^11^ and the ablation of focal right atrial triggering mechanisms.^12^ Further efforts to reproduce MAZE surgical outcomes with less linear ablations were made with some success using progressively more complex linear ablations within the right and left atria.^13^ These techniques, apart from identifying triggers within the right atrium, were essentially attempts to reproduce the MAZE surgical outcomes.^14^ These methods also, however, only had limited success due to associated technical difficulties in achieving adequate transmural lesion formation with available ablation catheters, and in completely addressing AF triggers.

Many of the above early approaches stemmed from evidence indicating that linear lesions eliminated reentrant regions within the atrium that were responsible for sustained fibrillatory activity. By creating multiple linear lesions within the left and right atria, small anatomic regions were subsequently established, which were not capable of sustaining reentry.^15^ This theory was further supported by multiple lines of evidence that demonstrated that a critical myocardial mass, as well as single and multiple wave fronts of propagation, were necessary for sustained fibrillatory activity.^16–20^ However, the seminal advancement in the field of AF occurred when spontaneous, focal initiation of human AF was documented in 1997, and confirmed in 1998, with the observation that ectopic beats from pulmonary veins triggered paroxysmal AF.^21,22^ This discovery ushered in the modern era of performing pulmonary vein isolation (PVI) for the treatment of paroxysmal, and later, persistent, AF.^23^ Furthermore, this methodology has been firmly elucidated to allow for longer-term freedom from AF in a majority of patients, without the need for extensive linear atrial ablation.

AF ablation was performed historically using non-irrigated 4-mm and 8-mm catheters. This catheter technology had several limitations, including the risk for tissue or electrode overheating, which could result in catheter char, coagulum formation, or steam pop. The development of char and coagulum posed a risk to lead to thromboembolic complications, while steam pop could result in cardiac perforation. In attempts to improve catheter safety, ablation was most commonly performed in a temperature control mode, which allowed for a reduction in power output to maintain a maximum allowed electrode temperature. Unfortunately, this restriction manifested as a suboptimal single-procedure freedom from AF of approximately 50%.^24,25^ In part, these outcomes were likely related to insufficient lesion formation resulting from inadequate power delivery. In addition, several factors fundamental to lesion formation could not be assessed or modified with early-generation catheters, such as convective heat loss, the above-mentioned coagulum formation (which would further limit power delivery), and catheter-tissue contact force. As a result, ablation time was often the only parameter under direct and measurable control of the electrophysiologist. Such was often insufficient to secure an optimally successful procedure, as biophysical studies of radiofrequency (RF) ablation have demonstrated that lesion formation is nonlinearly dependent on four critical factors: ablation time, power, catheter myocardial contact force, and convective heat loss.^26,27^ Only allowing for the control of ablation time leaves these other factors not well-addressed, thus potentially resulting in inadequate lesion formation and procedural failure.

### Efforts to address factors affecting lesion formation

The development of externally irrigated ablation catheters was a significant advancement and obviated several of the deficiencies inherent to standard, non-irrigated catheters. Initial irrigation was predominantly accomplished via irrigation holes within the distal aspect of the ablation electrode. Despite affecting only this segment of the electrode, there was significantly improved cooling at the electrode–tissue interface, where overheating is most common. This reduction in catheter overheating led to improved power delivery, as well as reduced char and coagulum formation at the distal electrode.^28,29^ Consequently, improved lesion formation was possible, and was associated with improved procedural success, in comparison with that seen with the use of standard 4-mm catheters.^30^

Limitations, however, remained with first-generation externally irrigated catheters. To achieve adequate electrode cooling, these catheters required substantial fluid delivery, a stipulation often leading to clinical volume overload states following long procedures. Additionally, first-generation designs cooled only the distal portion of the ablation electrode, leaving them vulnerable to char formation at the proximal portion. Second-generation externally irrigated ablation catheter designs improved on both these deficiencies by increasing the number of irrigation holes and spreading them more completely over the entire electrode surface area. This technology decreased the risk of char formation at the proximal portion of the electrode and allowed for a reduction in the needed amount of fluid delivery, due to improved electrode cooling.^31^ These advancements in external irrigation allowed for consistent power delivery even in myocardial locations such as the coronary sinus, where first-generation catheters often received only limited power delivery due to overheating.

Despite the improved design, the measurement of contact force (an important biophysical measure) was not addressed and, without this knowledge, lesion formation was neither consistent nor reproducibly achieved. Surrogate methods to assess lesion formation, such as impedance drop and electrogram elimination, were therefore commonly employed and were found to be helpful.^32,33^ However, these measures were not reliable predictors of lesion development, as they often correlated with the unmeasured contact force, or were dependent on confounding factors such as catheter orientation. This shortcoming led to the next significant advance in catheter design: the advent of contact force-sensing catheters allowed for the direct measurement of catheter-myocardial contact and directionality. More recently, this important development has translated to an improved success rate for ablation of AF,^34,35^ likely due to an improvement in lesion formation. Additional evidence from two contact force-sensing trials, SMART-AF and TOCCASTAR, demonstrated that when optimal contact force is used a significantly improved freedom from AF rate occurs, in comparison with that seen with the use of suboptimal contact force (81% versus 66% in SMART-AF; 75.9% versus 58.1% in TOCCASTAR).^36^ Using a combination of ablation time, consistent power delivery via second-generation irrigated catheters, and contact force measurement, adequate lesion formation was more reliably achieved.

In spite of this improvement, however, lesion formation continues to be inadequate in certain circumstances, and there remains an unmet need for accurate lesion measurement or software-based prediction methods. Evidence from the chronic assessment of PVI has demonstrated that even with the use of contact force catheters, frequent pulmonary vein recovery still occurs.^37,38^ To better improve chronic PVI outcomes, the direct measurement of lesion formation, including depth and diameter, would be optimal, although it is somewhat technically challenging, and there is no method that is clinically available or United States Food and Drug Administration (FDA)-approved at this time.^39–45^ Methods to achieve practical lesion measurement are under active research, but have not reached a level at which they are suitable for deployment in an electrophysiology laboratory. This absence has led to interest in software algorithm methods to predict or estimate lesion formation. These include nonlinear algorithms that utilize contact force, ablation time, and power settings.^46^ Such methods have been shown to predict lesion size in animal models,^47,48^ and are under investigation for their applicability to humans. Perhaps the use of such technology may allow for the accurate prediction of lesion formation, and could offer a consistent and operator-independent method to improve procedural success rates.

The final element affecting lesion formation is convective head loss and its effect on ablation-induced tissue temperature. Current-generation catheters are unable to monitor for heat loss or to measure tissue temperature in any form. A surrogate for convective heat loss may be the accurate measurement of myocardial tissue temperature during RF ablation. As myocardial cell death occurs above 50°C, the evaluation of tissue temperature would allow for the electrophysiologist to measure convective heat loss in a surrogate manner. The next step in catheter development is likely to focus on such measurements.

An alternative method to achieve lesion formation is ablation based on tissue cooling, or cryoablation, which leads to myocardial cell death due to both a direct cryothermal affect and microvascular destruction.^49–53^ This methodology was originally developed in the form of a 4 mm ablation catheter, where the ablation electrode was cooled to achieve sufficient myocardial tissue destruction. Originally used for the ablation of the atrioventricular node slow pathway for atrioventricular nodal reentrant tachycardia, this technology was later deployed for PVI for AF. Cryoablation was deemed less likely than RF ablation to lead to atrioesophageal or esophageopericardial fistula formation, although these conditions have since been described in case reports. The use of 4- or 5-mm electrode cryoablation catheters for PVI was adopted at several centers that were attempting to minimize fistula formation. Ultimately, however, this method proved too time-consuming for incorporation as standard use, and the efficacy for PVI achieved was not optimal in comparison with that of externally irrigated RF ablation. Next-generation cryoablation for PVI was achieved through the development of balloon-based technology during which the balloon was positioned at the pulmonary vein ostium and PVI was performed in a single step. This led to an improved procedure efficiency rate in certain centers.^54^ Of note, the STOP-AF trial evaluated the efficacy of cryoablation and demonstrated a 12-month freedom from AF rate of 69.9%.^55^ In this study, 11.2% of patients had phrenic nerve paralysis, with 1.5% of cases persisting beyond 12 months. A comparison of RF ablation using externally irrigated catheters versus cryoablation in the FIRE AND ICE trial demonstrated non-inferiority of cryoablation.^56^ However, notably, for the RF ablation arm, this trial used a mix of first-generation noncontact force and second-generation contact force catheters, a potential study limitation. Freedom from AF was observed in 64.1% of subjects undergoing RF ablation and in 65.4% of those undergoing cryoablation. This is somewhat lower than the rates observed in the SMART-AF trial using contact force-sensing catheters alone, in which an overall freedom from AF rate of 72.5% was demonstrated.^38^ Additional 30-month freedom from AF data supported the non-inferiority of cryoablation, in comparison with that of RF ablation.

Optimal use of either cryoablation or RF ablation is important to achieve a higher procedural success rate. When contact force was kept within an operator-selected range ≥ 80% of the time, 12-month freedom from AF in the SMART-AF trial improved to 81%, in comparison with 66% when the contact force was within range ≤ 80% of the time.^38^ In several studies, the use of second-generation cryoablation has also demonstrated a higher success rate and improved procedural efficiency than did first-generation cryoballoon ablation.^54,57,59–61^ Specifically, in a meta-analysis of 2,363 patients undergoing ablation using a second-generation cryoballoon, the one-year freedom from AF rate was 82%.^61^ Cryoablation though has a potential economic barrier due to the increased cost that results when additional catheters are used to treat concurrent atrial tachycardias.

Alternative methods for achieving PVI have been developed, but are investigational in nature and are not currently FDA approved. These include balloon-based technologies that incorporate laser or RF energy or circular multielectrode ablation catheters. In the HotBalloon study, a prospective multicenter study of balloon RF ablation, freedom from AF was observed in 59% of the ablation group.^62^ However, complications of pulmonary vein stenosis were observed in 5.2%, and transient phrenic nerve paralysis in 3.7%. The HeartLight^®^ (CardioFocus, Marlborough, MA, USA) laser-based balloon ablation system was also evaluated in a multicenter study of 353 patients.^63^ This study demonstrated a promising freedom from AF rate at one year of 61.1%, which was equivalent to that seen in the RF ablation arm. Diaphragm paralysis was observed in 3.5% of patients, though no pulmonary vein stenosis was noted. In regards to circular multielectrode catheters, there were safety concerns regarding non-irrigated catheters, as an analysis that compared the performance of either external irrigated RF ablation or cryoballoon demonstrated a 1.48 times higher risk of silent cerebral ischemic lesions (identified via post-procedural magnetic resonance imaging (MRI)).^64^ Next-generation catheters have effectively reduced this risk. The nMARQ™ catheter (Biosense Webster, Diamond Bar, CA, USA) is an irrigated circular ablation catheter that has demonstrated a one-year success rate ranging from 46% to 87% (with the three largest trials yielding rates of 65%, 73%, and 75%, respectively).^65–76^ Another example, the pulmonary vein ablation catheter (PVAC; Medtronic, Minneapolis, MN, USA) sports a non-irrigated design with an improved safety profile for thromboembolic events. This catheter has demonstrated one- to two-year success rates ranging from 38% to 86% (with the three largest trials yielding rates of 47%, 65%, and 75%, respectively).^77–91^ There is some, though limited, data available on the achievement of improved procedural success rates using multielectrode ablation in comparison with using single-electrode irrigated ablation.^92,93^

Importantly, despite the early mixed success rates for these emerging technologies, these studies typically represented first- or early-generation devices that had likely not reached full maturity or efficacy. The goal of performing longer or larger contiguous lesions with a single ablation procedure remains attractive, especially for its potential to improve procedural efficiency.

Another method to improve outcomes with ablation has focused on electrical isolation of the left atrial appendage (LAA). There has been interest particularly involving this subject and longstanding persistent AF, where the LAA may be a source of AF rotors or focal drivers. This concept was tested in the BELIEF trial, an open-label randomized trial of 168 patients with longstanding persistent AF.^94^ In this study, patients underwent either extensive atrial ablation alone, or such in combination with electrical LAA recurrence following a single procedure, in comparison with 28% in patients with no isolation performed. This improved outcome was replicated and expanded in a study comprised of 90% non-longstanding persistent AF, where 200 consecutive patients underwent cryoballoon PVI alone or in combination with LAA isolation.^95^ Following 12 months of follow-up and an average 1.2 procedures, the group who underwent PVI alone had a 67% freedom from atrial tachycardia, versus 86% when appendage isolation was also performed. Further studies will be needed to assess whether these results are widely applicable, and whether the potential increased risk for appendage thrombus formation following isolation is acceptable.

The superior vena cava (SVC) is another potential trigger for AF, and in a seminal study, was found to account for 6% of paroxysmal AF trigger sites.^96^ In one study, SVC isolation was evaluated as an adjunct to PVI in a group of 320 consecutive patients consisting of both paroxysmal and persistent AF.^97^ Patients with either longstanding persistent or persistent AF had no improvement in freedom from atrial tachycardia. However, in paroxysmal patients, 90% of those with SVC isolation were free of atrial tachycardia, versus 77% of those without isolation. This result, however, could not be replicated in other randomized studies.^98^ In addition, SVC isolation has the potential to lead to complications such as SVC stenosis and sinus node injury. Therefore, currently recommendations are to proceed with SVC isolation only when tachycardia, frequent ectopy, or AF is documented to originate from this structure.

The intrinsic cardiac autonomic system located in the ganglionated plexuses (GP) has also been shown to participate in the initiation and maintenance of AF.^99^ Stimulation of the GP results in parasympathetic stimulation leading to early after depolarizations, calcium transient triggered firing in the pulmonary veins, and initiation of AF. The GP are located near the left superior and inferior pulmonary veins, the right superior and inferior pulmonary veins, and the Marshall tract. In a randomized study of paroxysmal AF, pulmonary vein isolation alone or in combination with GP ablation was performed. In this study, single-procedure freedom from recurrence was 46% with PVI alone versus 73.5% with PVI and GP ablation.^100^ This strategy was particularly beneficial when PVI was performed ostially. Current ablation techniques focus on pulmonary vein antrum isolation, which encompasses the ablation of the GP locations inadvertently. This additional GP ablation may account for why antral ablation has a higher success rate than ostial PVI. As a result of the wider ablations performed, most centers no longer ablate the GP as a separate step in the procedure.

## Mapping developments for non-pulmonary vein AF drivers

PVI remains the cornerstone in the ablation of both paroxysmal and persistent AF. In paroxysmal AF, PVI alone can achieve reasonable success rates, as mentioned above, of approximately 80% with contact force RF ablation, or second-generation cryoballoon ablation. PVI alone in persistent AF has not proven as successful. For this reason, intensive research has been performed for methods to map non-pulmonary vein drivers. Ablation of these additional sites holds promise to improve ablation in persistent AF, as well as to identify the subset of paroxysmal patients with non-pulmonary vein triggers in whom additional ablation may be helpful. Prior studies have indicated the importance of several non-pulmonary vein triggers arising from locations such as the SVC, the vein of Marshall, the coronary sinus, the crista terminalis, and the posterior left atrium. The incidence is as high as 20% in paroxysmal AF and 35% in persistent AF, respectively.^101–103^ The identification of these triggers has proven to be important, and is currently the preferred approach over substrate ablation alone.

Basic electrophysiology research has demonstrated the presence of a variety of non-pulmonary vein AF driver types, including microreentry, spiral rotors, and focal triggers, among others.^104–112^ These mechanisms have also been modeled with software capable of accurately reproducing the observations in high-density tissue and animal studies. The translation of these mechanistic findings into an effective ablation strategy that improves freedom from AF has been challenging. One of the largest randomized multicenter studies of persistent AF, the STAR-AF II trial, evaluated PVI only versus PVI plus linear ablation or complex fractionated atrial electrogram (CFAE) ablation. This study did not determine the occurrence of any significant improvement in freedom from AF with the addition of ablation beyond PVI. The overall single-procedure success rate in these persistent patients, however, was suboptimal at 59%, indicating a need for alternative ablation strategies to improve outcomes. Additional evidence has supported that ablation of CFAE has not produced consistent improvements in ablation outcomes.^113–120^ Further studies have indicated that multielectrode mapping may identify critical CFAE regions with greater accuracy than single-electrode catheter mapping by allowing for the separation of CFAE regions into either passive or driver regions.^121–125^ The use of multielectrode mapping has also been utilized to identify alternative non-pulmonary vein drivers such as regions of high spatiotemporal dispersion, with ablation leading to improved freedom from AF.^126,127^

Two additional methods to map non-pulmonary vein drivers include surface electrocardiogram arrays and the FIRM mapping system (Topera Medical, Palo Alto, CA, USA). Studies evaluating these methods have identified sites consistent with spiral rotors and focal drivers of AF in humans for the first time without the need for incorporating surgically placed electrodes.^22^ There is significant interest in such methods, especially regarding their potential to identify critical electrophysiology-based sites.

The FIRM mapping system (Topera Medical, Palo Alto, CA, USA) utilizes a balloon catheter with 64 electrodes to map the entire atrium. This method involves using pacing to initiate AF, if it is not present at baseline, and placing the mapping balloon catheter within the left atrium. Proprietary software analysis of the electrograms through phase mapping, and analysis of repolarization and conduction dynamics, have been used to map patient-specific sources.^128^ Specific sites of non-pulmonary vein AF mechanisms are identified through electrogram analysis and marked on a corresponding map. This information is then extrapolated to an existing electroanatomic map, which has been created using an alternate mapping system. The ablation procedure typically would then involve isolation of the pulmonary vein antrum using standard contact force-sensing externally irrigated catheters, followed by ablation of the FIRM-identified non-pulmonary vein sites. Procedural endpoints include AF termination or average AF cycle length slowing.

Initial study results achieved with FIRM mapping were positive. The pivotal CONFIRM trial was a 92-patient multicenter study that found an intermediate six- to 12-month procedural success rate of 82% in a mixed cohort of paroxysmal and persistent AF.^129^ A follow-up multicenter study involving 78 patients demonstrated a single-procedure success rate of 87.5% again for a mixed paroxysmal and persistent cohort.^130^ In the initial studies, local rotors or focal impulses were detected in 97% of patients with an average of 2.1 sources. The sources identified were within the left atrium in 76%, which included pulmonary vein locations and disparate locations such as posterior, inferior, roof, and anterior regions. The remaining 24% of locations were within the right atrium in the inferolateral, posterior, and septal regions. Unfortunately, the reported positive initial results could not be replicated in other studies, including in a longer-term 18-month multicenter study involving 43 patients, of whom 37% had freedom from AF.^131^ The precise reason for these disparate results is not definitively established. Possibilities include either poor basket electrode-myocardial contact, leading to inadequate electrogram resolution sufficient for rotor site identification, or the presence of an underlying technical software deficiency that is limiting accurate identification of driver sites.

The CardioInsight mapping system (Medtronic, Minneapolis, MN, USA), a noninvasive body surface mapping analysis device, uses 252 external electrocardiogram electrodes in combination with computed tomography (CT) to create simultaneous biatrial three-dimensional maps. Specific algorithms, including wavelet transformation and phase mapping, are then applied to allow for the identification of AF driver sites.^132^ This system is currently available, but is in need of multicenter randomized-based evaluation to further assess its abilities. Additionally, non-invasive body surface mapping suffers from a potential hurdle in that it is not capable of precise localization to millimeter accuracy, as is standard when typical maps are created (eg, when using intracardiac catheters and current mapping systems).

Advancements in the accuracy of the left atrial map, from which AF triggers are identified and the ablation lesion set is planned, have been made as well. Historically, the left atrial anatomical map was created using a single-electrode ablation catheter and point-by-point mapping. This was an effective method, but was also time-consuming; a lack of a high-density anatomic point cloud also limited the completeness of the map. Progress in software and multielectrode catheters allowed for a progression to occur, from the use of point-by-point maps obtained with single-electrode catheters to multielectrode mapping, with multiple simultaneously acquired points. Additionally, the increasingly widespread use of preprocedural atrial CT or MRI, and maps derived from intracardiac echo, have improved atrial anatomic mapping. One method to improve procedure efficiency and accuracy is to merge a preprocedure left atrial CT scan or MRI scan onto a basic and incomplete left atrial anatomy image acquired via use of a mapping catheter.^133–135^ This technique allows for a complete left atrial map to be generated in a relatively efficient manner.

Beyond map creation, preprocedural imaging has also proven useful to ensure that complete anatomic mapping is accomplished. Reducing inaccurate anatomy has led to improved circumferential PVI.^136^ Other benefits to imaging include evidence for reduced radiation dosing and improved outcomes, in comparison with those achieved using standard point-by-point mapping.^137,138^ A second method for imaging the left atrial anatomy is by intracardiac echo.^139^ Using echo images, the endocardial contours are traced within the mapping system, allowing for a complete left atrial anatomy map to be developed without the need for catheter manipulation within the chamber of interest. Intracardiac echo, as well as the CT and MRI merge technique, can provide a complete left atrial anatomy when the mapping catheter cannot be maneuvered to achieve such a result.

Multielectrode mapping of the cardiac chambers using rapid and automatically acquired anatomic points has proven to be accurate and effective for rendering a complete model of a patient’s atrial anatomy.^140^ This technique is currently one of the most commonly utilized, as it provides accurate mapping in a relatively efficient manner. This can be performed using either a circular or multispline catheter. In addition to anatomy, this technique can generate high-density voltage maps, which provide additional useful information about the substrate for AF drivers and other scar-mediated arrhythmias.^141^

## Future directions in the ablation of AF

There has been intense research done into mapping non-pulmonary vein drivers to improve procedural efficiency and outcomes. PVI alone has proven to be reasonably effective for the treatment of paroxysmal AF, but remains somewhat suboptimal in cases of persistent AF. Methods to map non-pulmonary vein sources of AF will likely prove important in the future to achieve single-procedure success rates for both paroxysmal and persistent AF, similar to other atrial arrhythmias. Technologies such as FIRM or body surface mapping to identify AF drivers hold great promise but also potential technological deficiencies. A lack of spatial accuracy, and difficulty with electrogram resolution due to inadequate electrode contact, may account for the mixed results achieved using these methods. Basic research has been consistent in identifying that such drier sites should be present in humans, and therefore there is reason to believe such mechanisms can be identified and ablated to improve procedural outcomes. The next phase in AF mapping will depend on developing mapping techniques that are accurate in all aspects, temporally, spatially, and with high electrogram resolution.

Heterogeneous lesion formation remains another potential key limitation, and has likely confounded studies like STAR-AF II, and techniques such as FIRM-based ablation. Until methods are developed to assess lesion formation, either through software or direct measurement, PVI and ablation of non-pulmonary vein sites are subject to interoperator variability. This variability often manifests as disparate results between centers, as has been observed in many trials to date. These findings do not definitively negate the importance of such non-pulmonary vein sites, but likely underline a fundamental technologic impediment. Ultimately, reproducible mapping of alternative AF driver sites, in addition to consistent, non-heterogeneous lesion formation, will be necessary to achieve higher single-procedure freedom from AF.

Successful ablation of AF ultimately relies on three fundamental and equally important factors. First, (1) a reproducible and measurable method to achieve lesion formation in the desired location must exist. Additionally, (2) an ability to map and understand the basic mechanisms and optimal ablation sites to eliminate AF, and (3) an accurate anatomic map from which to perform electrogram and ablation localization, are also ideal. Current mapping systems using multielectrode catheters can produce anatomically accurate cardiac chambers. RF ablation lesions have improved with the current generation of contact force external irrigation catheters. Further methodology to predict lesion formation through software-based algorithms is the next generational step in achieving consistent and reliable lesions. Catheter development, which will lead to accurate measurement of tissue temperature as a surrogate method to assess for convective heat loss, will likely further improve consistent lesion formation. Mapping of non-pulmonary vein driver sites is under active development, with several methods currently available, but with limitations that prevent widespread utility. Ultimately, future success of any such strategy depends heavily on advancing fundamental knowledge of these mechanisms in humans, and the existence of the ability to accurately map such sites with both high spatial and electrogram resolution. With further advancement in these three areas, the ablation of both paroxysmal and persistent AF will become a reliable, reproducible, and electrophysiologic-guided, rather than anatomically based, procedure.

## Conclusions

Significant advancements have been achieved in the ablation of AF over the past 20 years. Current state-of-the-art ablation technology can achieve a high success rate for ablation and paroxysmal AF. Further technological advancements such as lesion prediction and catheter developments that accurately measure tissue temperature will further improve the rate of success. These developments will be especially important for the ablation of persistent AF where all elements—from accurate AF driver mapping to consistent lesion formation—are fundamental to a successful result.
